# Testing the sorption of economic metals from seawater on waste biomass: a preliminary feasibility study

**DOI:** 10.1007/s10967-026-11163-w

**Published:** 2026-09-23

**Authors:** Steven McGowan, Claude Degueldre, Farid Aiouache, Hao Zhang

**Affiliations:** 1 Dept of Engineering, Lancaster University, Lancaster, UK; 2 Lancaster Environmental Centre, Lancaster University, Lancaster, UK

**Keywords:** Seawater, Uranium capture, Biomaterials, Absorbents, Partition co-efficients, Biofilms

## Abstract

The development of the modern and sustainable era of humanity has been built on continuing reliance of many scarce resources. These are primarily extracted from limited geological sources, with estimated capacity in the decade to century ranges. It is also possible to extract them from the saline environment. These exist in near constant concentration, in equilibrium between the influx by geographical erosion, and the deposition into the sea floor. This work aimed to report efforts to quantify the performance of methods intended to absorb these resources using low cost biomass adsorbents. These were compared against materials in a nano-cellulose membrane. The nanocellulose proved to be a significant impediment, despite literature to the contrary, but did improve our understanding.

## Introduction

Over the past decade, renewed interest has emerged in the novel sourcing of economic metals, driven by the identification of the limits of geological reserves, and the rising demand for technology metals [9]. These metals are used to fabricate the components in vehicles, consumer smart devices, and renewable energy sources [26]. There is a growing realization that advancing clean energy technologies and sustainable products will require substantial amounts of critical metals and otherwise valuable elements, including platinum group metals, lithium, copper, cobalt, silver, and gold [12]. However, most of the materials are still produced almost exclusively by through mining, extraction, and processing of geological mineral ores [26]. These traditional processes have high environmental footprints and a significant legacy of complex remediation challenges for future generations [29]. For certain minerals, a potential alternative sourcing route lies in extracting these elements from natural erosion streams, specifically at their endpoint, where they become dissolved in seawater [38].

Natural erosion of geological materials continuously releases dissolved elements from the land into the sea, carried in rivers and other surface flows. These elements exist in equilibrium, balancing deposition in seafloor deposits with the influx from the geological sources [38]. Functionally, these would represent a potentially renewable resource, as at least some of the mineral demand would be far below the magnitude of these geological fluxes [8]. Most extraction approaches are based on selectively extracting a particular element onto a specific media which can be recovered, either directly from the seawater or from brine, the concentrated by-product of desalination [5].

Extraction strategies can target either single elements, such as lithium [35] or uranium [7], or multiple elements [18]. Most existing approaches rely on synthetic membranes to either sorb or filter the desired elements, but these often involve high synthesis costs [9] or significant energy requirements [11]. An alternate approach, initially developed for wastewater clean-up, involves using by-product biomass as a filtrant. Such materials are cost-effective, abundant and possess high surface capacities, improving recovery efficiency [15, 22, 33].

These approaches have previously demonstrated that, in the right conditions, it is possible that we could generate sufficient sorbance to be economically viable, but the precise nature of those conditions have not been investigated sufficiently, nor has there been a consistent methodology applied to establish validation of these conditions. Each approach has been conducted with different options for criteria, and some of the outcomes are predisposed on factors which are not reported in a consistent fashion.

The initial testing in the previous work has begun establishing some of these requirements, such as use of natural waters, particle size, but this also established some improvements that were necessary. Furthermore, those materials evaluated represent only an exceedingly small subset of all possible potential sorbents, and was focused on only a single element.

This work aims to expand our understanding of the potential of these further materials while continuing to evaluate our approach to their sorptive behaviour.

## Theory

Free cations in natural conditions can have a wide variety of shapes, and can form coordinations with naturally occuring ligands, and over the the full range of critical minerals, has a high feasibility of forming mixed ligand complexes.

The prevalent (simple) ligands with strong affinities for these cations in saline environments are Cl^−^, OH^−^, CO_3_^2−^, HO_2_^−^ and SO_4_^2−^ [10]. The relatively near-neutral-to-weakly-alkaline (pH 8.0 *E*(*h*) 0.8 V) nature of seawater is the primary driver on selection, as these ligands are pH sensitive, driving the relative concentrations in solution, and concequently, their relative cumulative partition co-efficients which exhibit a dense buffering effect, due to the median conditions being controlled by the dominance of seawater by volume, produces relatively fixed prevalences. These pervalences can be altered though modification of pH, or redox potential (*E*(*h*)) of the liquid (McGowan, et al., 20, McGowan, et al., 21).

In contrast, the natural sorbents as biological organic materials, exist on a sliding scale, between wholly polyphenolic, and wholly carboxylic polysaccharides. However, both materials are based on a similar sized replicating unit for lignin or hesperidin in polyphenolic compounds, and cellulose or demi-cellulose for polysaccharide, due to the mechanical processes in synthesis in biomaterials [4]. These two serve as sorbent sites, producing a net-sorbent capacity, which will also vary by pH and *E*(*h*) (Zhang, et al., 37, Yang, et al., 36).

These processes are dynamic, complex, interlinked equilibriums, limited by many factors. In represent these natural conditions, it is necessary to replicate them as far as possible in the tests, through use of natural seawater, and natural residues that are as unaltered as possible.

## Experimental

The experimental procedure followed that previously reported in reference [22] with several changes based on the review of the results.

The seawater samples were collected from Trafalgar Point (54.075192 N, − 2.878758 W), Stone Jetty, Morecambe, NW UK, on the Irish Sea at high tide, over 8 samplings and were pooled into a common tank, and allowed to equilibrate, which collectively reported salinity of 3.2% and a density of 1.018 g mL^−1^.

Where referred to, deionised water is Suprapure© Quality. The nitric acid is prepared using this water, and 64 ml of Aldrich© 70% redistilled, ≥99.999% pure nitric acid, in a 500 ml volumetric flask.

Five new sample materials were analysed along with the best-performing samples from the previous screening, which served as inter-batch controls. The returning samples were Kale, Garlic Powder, Orange Peel, Peanut Shell, Potato skin, were included due to previous strong performance [22].

The samples were air dried using a Aicok© Digital Dehydrator at 50 °C for 72 h, then ground using a mechanical rotary grinder, then a sieve was used to partition the particle size, limiting the particle size to 0.2 mm, with oversized particles being returned to the grinder, until they meet the size requirement.

Coffee Grounds (the insoluble components of a commercial blend of *Coffea arabica and Coffea Canephora*), red tea (*Aspalathus linearis*) and blackberry (*Rubus fruticosus*) were identified due to their wide availability and antioxidant properties [1, 6, 24]. White Birch bark (*Betula pendula*), noted for its high phenolic content, strong antioxidant activity, and history of use in land remediation where the bark acts as a reservoir for heavy metals during bioaccumulation, was also included [13]. Spring greens, which are a mixture of commercially available green vegetable matter (primarily various *Brassica)* were chosen for their high antioxidant and iron contents and for their similarity to Kale, which has been investigated previously.

All materials were sourced locally to Lancaster University, except for the white birch bark, which was supplied by Forestry and Land Scotland from routine felling operations at Carsewood Park (55°48′26.2"N 4°33′22.3"W). The drink residues (blackberry, red tea, and coffee grounds) were soaked in deionised water for 4 h to remove soluble components, before the drying.

The number of replicates was increased to 3 + 1 for the first phase and 4 + 1 for the second, to improve result reliability and to allow final pH measurement without contamination risk.

The range of elements assessed for this study was expanded. Considering the list of elements reported in Table 1 and the practicality for this approach, lithium was excluded, as its capture in sodium- and magnesium- environment would not yield significant sorption [14]. Furthermore, a reference standard for niobium was unavailable, so this was also discounted, although it was not expected to be significant. The calibration standard used included cobalt, but in practice, the concentrations of cobalt consistently fell below the limit of detection (LoD) of the instrument and were therefore discounted. However, since the calibration standard included manganese as a co-standard, manganese was included among the elements of interest. Therefore, the elements analysed were vanadium (V), manganese (Mn), nickel (Ni), Copper (Cu), molybdenum (Mo), gold (Ag), silver (Au), and uranium (U).

**Table 1:** Geological reserves of minerals of economic interest, and the corresponding reserves in seawater dilution in 2022

Element	2022 terrestrial reserves (× 10^6^ tonnes)	2022 world production (× 10^6^ tonnes)	2022 reserve supply ratio (years)	Seawater concentration (mg/L)	Dissolved metals in seawater (× 10^6^ tonnes)
Cu	0.89	0.026	34	0.0009	1170
Ni	1000	3.3	303	0.0066	8580
V	26	0.1	260	0.0019	2470
Mo	12	0.25	48	0.01	13 000
Li	98	0.13	754	0.178	231 400
Co	25	0.19	132	0.00039	507
Nb	17	0.079	215	0.000015	1.3
Ag	0.052	0.0031	17	0.00028	364
Au	0.55	0.026	21	0.000011	14.3
U	6.078	0.049	124	0.0033	4290

Terrestrial data from (National Minerals Information Center, 25) except for Uranium (World Nuclear Association, 34) Seawater Concentrations and reserves from (Turekian, 32), and [9]

Review of these elements, and the potential interference effectins in ICP-MS from elements present in standard concentrations in seawater, some known issues of interference are present [31].

The main common impacts are 51-vanadium, where there are two potential interferences: [^35^Cl^16^O]^+^ and [^34^S^16^OH]^+^, and 55-manganese, which has a known interferent in the form of ^32^S^23^Na^+^ [31]. These result in low degrees of sensitivity exhibited in the relevant results.

Nickel (^58^Ni and ^60^Ni) also has interference from a number of low frequency isotope pairings in NaCl but these are <10% by weight, so the occurrance of these intereferents has a cumulative frequency of less than 1% [2].

Outside these, as listed, interfering co-elements are mostly radioactive with short half-lives, and are very scarce to non-existent in seawater, or of poor electron stability (for example, [BrO]^−^), so may be discounted.

Once the samples were prepared, a precise weight (varying by density of material, but usually ~1 g) was placed into a 50 ml PTFE sample tube, and a precise weight of seawater was added (approximately 40 g).

In addition, for the second phase, the biomaterials were placed into nanocelluolose filter bags. As with the materials, these were accurately weighed before addition of the sample, so the weight can be discounted. The manufacturer (Ppanda) described these as ash-free nanocellulose membranes with a reported pore diameter of 20 μm, which was comparable to the filters previously used and, according to literature, expected to retain the materials, without affecting ion transport. [19]. However, as discussed in the results section, these claims appear to be inaccurate in practice.

The samples were left in contact with the sea water for 24 days.

Further to this, control samples of the seawater were placed into the same tubes, without any biomass present (but did include the filter bags for the second batch), to act as system controls, to establish if there were any losses in processing.

One sample was separated, and the liquid phase were tested using a HANNA edge pH meter.

The measurement samples had a volume of 10 mL of the aqueous phase extracted using a mechanical pipette and digested in 10 mL of heated 2 M HNO_3_. This was then filtered using a Whatman™ 0.4 µm PTFE membrane filter, then made up to 50 mL, using a clean volumetric flask. The solution was then transferred to a clean tube for storage.

For the first batch, the remainder was filtered using a Whatman™ 595 150 mm filter paper to capture the solids, then the filter paper and residues were digested in 20 mL of heated 2 M HNO_3_.

For the second batch, the filter bag was carefully retrieved, and dried in the air dryer for 72 h at 50 °C, then was weighed to establish the new weight, before being digested in the same fashion.

The resultant digestate was then filtered through a Whatman™ 0.4 µm PTFE membrane filter then made up to 50 mL, using a clean volumetric flask. The solution was then transferred to a clean tube for storage. In addition to the samples themselves, the blank and control samples were also subjected to the same process.

All samples were then diluted by a factor of ×16 with deionised water, to minimise the impact of salt precipitation on the ICP-MS instrumentation.

The first batch was analysed by Prof. Martine Leermakers and Prof. Yue Gao in AMGC Group, Vrije Universiteit Brussel (VUB), Belgium, using a PLASMA 3 MC-ICP-MS (ERC Lumière).

Samples for the second batch of samples were analysed by ICP-MS at Lancaster Environmental Centre Trace Metals laboratory using a Thermo-Fisher Scientific Series X7.

A common multi-standard was used in all ICP-MS. For the ICP-MS, the mass scan range was set for standardisation on a range as listed along with the Limit of Detection calculated from the zero controls and calibration standards, in the table below.

## Results and discussion

**Fig. 1:**
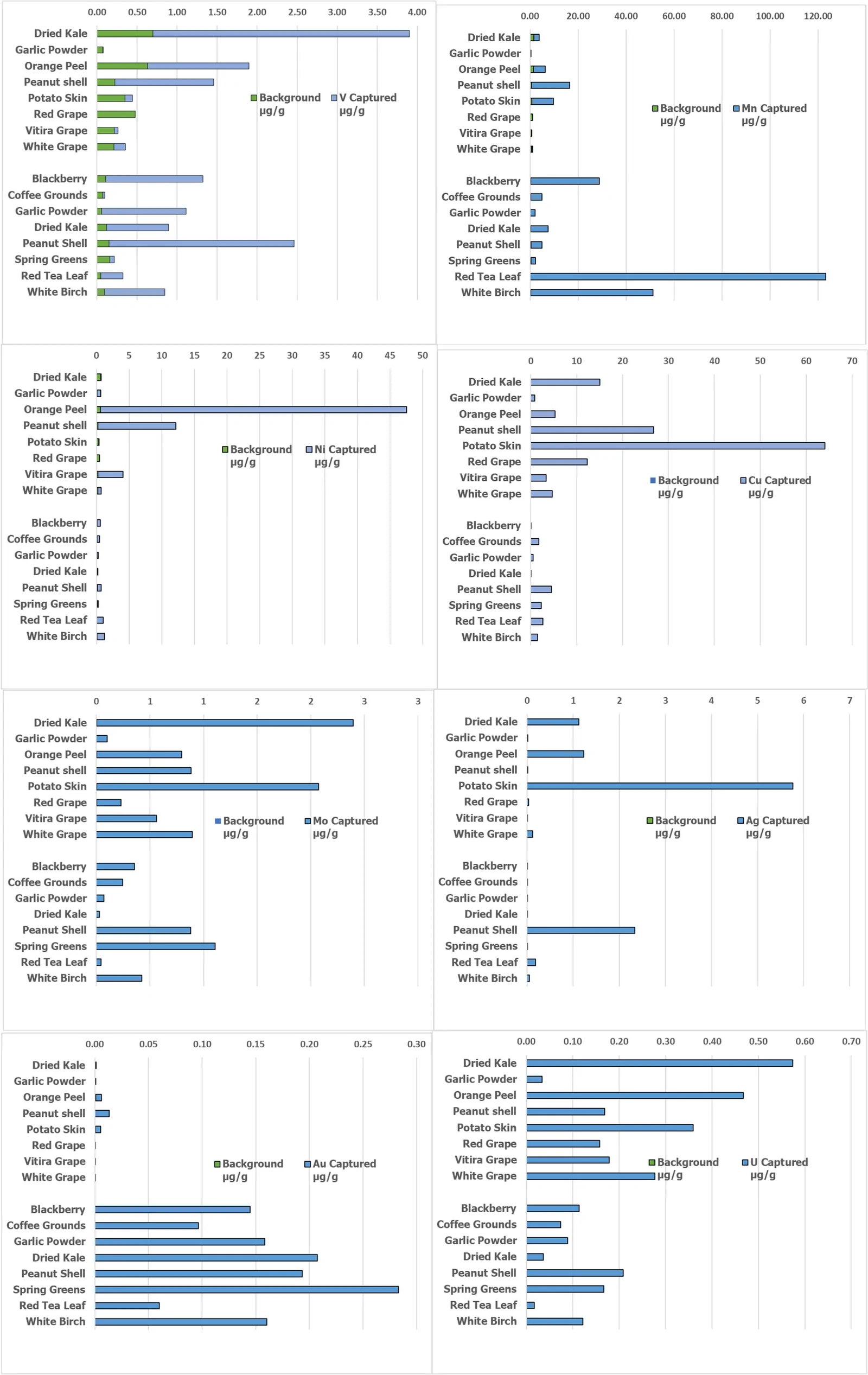
Metal concentrations in various biomass samples, before and after contact

From the samples analysis by ICP-MS, a number of results were identified. Seawater was reviewed and contained an average uranium content of 2.96 ppb and 3.57 ppb respectively, consistent with reported values in the previous paper. Results of these analysis are presented in Fig. 1 as split graphs and numerically in Table 2. Table 2 also includes the total mass balance as a percentage (%).

**Table 2:** Isotope and limit of detection anaylsed for ICP-MS

Element	Limit of detection (LoD) μg.l^−1^ (ppb)
51 V	1.562×10^–1^
55 Mn	3.651×10^–1^
60 Ni	1.416×10^–1^
65 Cu	5.860×10^–5^
95 Mo	7.884×10^–4^
107 Ag	6.354×10^–5^
197 Au	1.853×10^–04^
238 U	2.908×10^–5^

Generally, all materials exhibited at least weak sorption, with several strong exceptions, detailed below. However, significant discrepancies were observed between the samples enclosed in in bags and those in direct contact with the liquid, with the bagged samples showing consistently lower rates, suggesting that the filter material acted as an interferent. Nickel, copper, and manganese exhibited the highest levels of sorption among the target elements. As these elements are biologically significant their selective binding to intrinsic biomolecules is expected [34].

No element achieved sufficient sorption to produce concentrations comparable to ore-grad materials, which was expected in a preliminary test. Where strong sorbance was exhibited, a rate of 80–90% removal was the norm, indicative of limitation by the small liquid volumes and depletion of the dissolved phases, rather than capacity, resulting in measured concentrations close to, or in the case of V and Ni, under, the instrument limit of detection (LoD) in the liquid phases. Where liquid phase concentrations were below the lowest Standard, but above the LoD it was necessary to extrapolate the Standard curve, and reported as both the LoD (“Measured”) and as the Extrapolated (“Estimated”) values. The total masses of available elements between batch units were highly variable as summarized in Table 3, primarily due to the detection limit of the ICP-MS, as even its high sensitivity proved insufficient for accurate measurements.

**Table 3:** Average concentrations, and sorbance partition of material post capture

	V surface conc µg.g^−1^	V Sorbed measured /estimated (%)	Mn surface conc µg.g^−1^	Mn sorbed (%)	Ni surface conc µg.g^−1^	Ni sorbed measured /estimated (%)	Cu surface conc µg.g^−1^	Cu sorbed (%)
Dried kale	3.9±0.06	54% /86%	3.65±2.76	14	0.6±0.08	3% /3%	15.11±5.19	12%
Garlic powder	0.07±0.01	17% /54%	0.17±0.02	2	0.64±0.42	2% /7%	0.94±0.71	7%
Orange peel	1.88±1.79	35% /56%	6.32±6.05	21	47.58±41.37	21% /32%	5.28±1.4	19%
Peanut shell	1.45±0.42	54% /69%	16.53±3.19	31	12.19±4.34	31% /43%	26.79±5.45	42%
Potato skin	0.44±0.06	17% /45%	9.75±0.77	51	0.4±0.06	51% /3%	64.04±16.57	70%
Red grape	0.36±0.1	17% /53%	0.83±0.24	6	0.32±0.09	6% /6%	12.35±4.09	16%
Vitira grape	0.25±0.06	21% /77%	0.5±0.21	13	4.05±2.76	13% /53%	3.37±1.23	30%
White grape	0.34±0.21	17% /73%	1.04±0.43	8	0.7±0.48	8% /21%	4.69±3.35	27%
Blackberry	1.33±0.06	30% /30%	28.89±2.76	25	0.61±0.08	25% /22%	0.09±5.19	3%
Coffee grounds	0.1±0.01	4% /4%	5.05±0.02	17	0.47±0.42	17% /3%	1.74±0.71	37%
Garlic powder	1.11±1.79	18% /18%	2.09±6.05	17	0.3±41.37	17% /1%	0.56±1.4	10%
Dried kale	0.89±0.42	13% /13%	7.51±3.19	17	0.21±4.34	17% /13%	0.36±5.45	7%
Peanut shell	2.46±0.06	39% /39%	4.94±0.77	24	0.69±0.06	24% /44%	4.54±16.57	45%
Spring greens	0.22±0.1	2% /2%	2.34±0.24	9	0.3±0.09	9% /22%	2.31±4.09	17%
Red tea leaf	0.32±0.06	17% /17%	123.26±0.21	25	1.03±2.76	25% /38%	2.69±1.23	66%
White birch	0.85±0.21	18% /18%	51.18±0.43	23	1.22±0.48	23% /63%	1.56±3.35	36%

	Mo surface conc µg.g^−1^	Mo sorbed (%)	Ag surface conc µg.g^−1^	Ag sorbed (%)	Au surface conc µg.g^−1^	Au sorbed (%)	U surface conc µg.g^−1^	U sorbed (%)
Dried kale	2.39±0.66	44	1.13±0.45	53	0±0	1	0.57±0.17	74
Garlic powder	0.1±0.03	21	0.02±0.01	46	0±0	7	0.03±0.01	59
Orange peel	0.8±0.17	47	1.23±1.72	41	0.01±0	10	0.47±0.07	84
Peanut shell	0.88±0.1	72	0.01±0	11	0.01±0	24	0.17±0.06	56
Potato skin	2.07±0.17	41	5.77±5.63	98	0.01±0	8	0.36±0.07	90
Red grape	0.23±0.32	16	0.03±0.05	13	0±0	1	0.16±0.15	54
Vitira grape	0.56±0.16	58	0±0	0	0±0	1	0.18±0.07	92
White grape	0.89±0.88	57	0.12±0.05	72	0±0	2	0.28±0.26	65
Blackberry	0.35±0.66	47	0.01±0.45	33	0.14±0	16	0.11±0.17	34
Coffee grounds	0.25±0.03	61	0±0.01	34	0.1±0	16	0.07±0.01	34
Garlic powder	0.07±0.17	7	0±1.72	17	0.16±0	15	0.09±0.07	20
Dried kale	0.03±0.1	1	0±0	17	0.21±0	15	0.04±0.06	11
Peanut shell	0.88±0.17	59	2.34±5.63	45	0.19±0	11	0.21±0.07	35
Spring greens	1.11±0.32	18	0±0.05	13	0.28±0	12	0.17±0.15	22
Red tea leaf	0.05±0.16	12	0.18±0	23	0.06±0	11	0.02±0.07	15
White birch	0.42±0.88	37	0.05±0.05	74	0.16±0	8	0.12±0.26	28

**Fig. 2:**
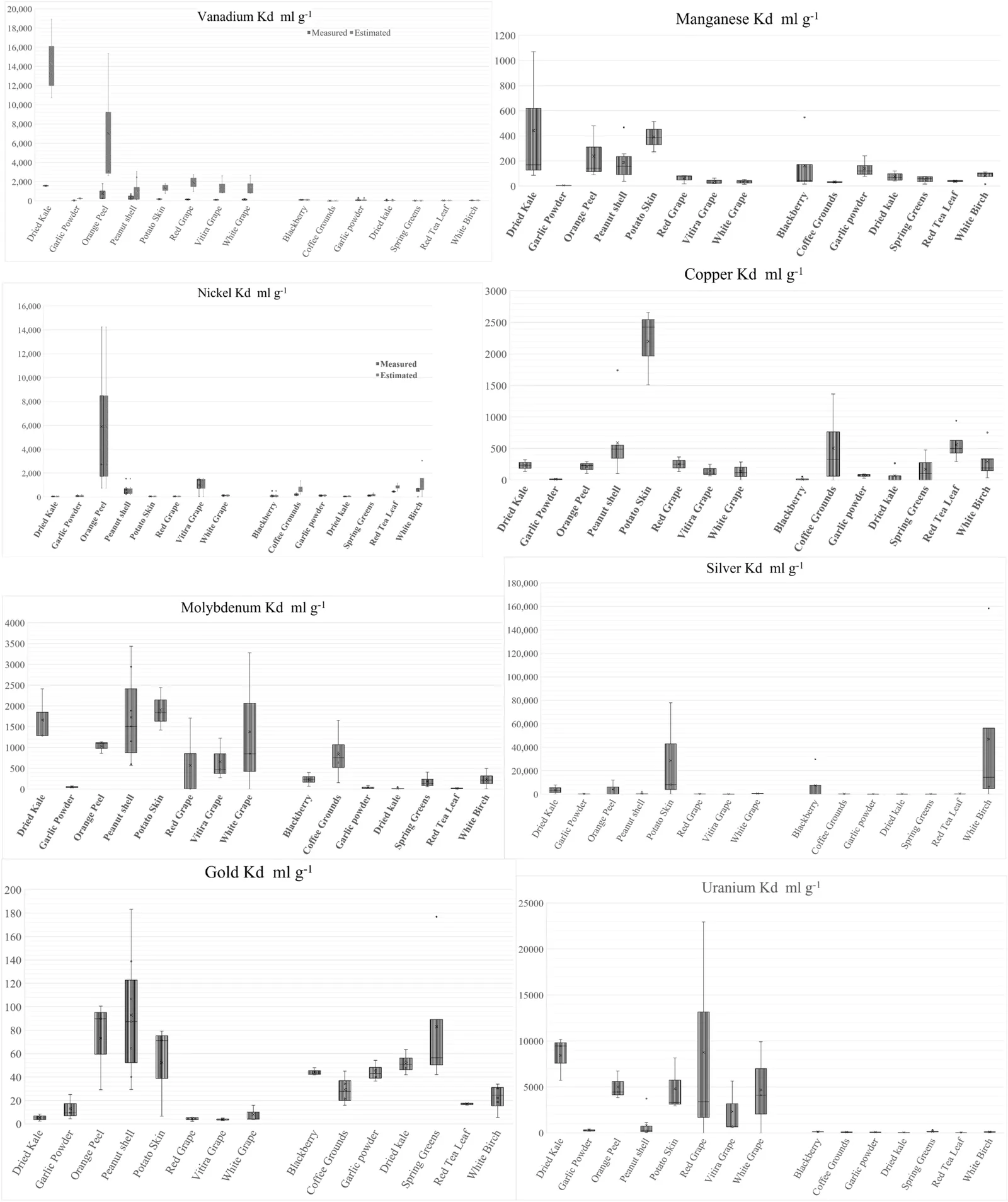
Partition coefficients *K*_*d*_ of various metal ions from seawater to biosorbants (ml g^−1^)

Across all elements of interest, potato skin was the most effective material, showing relatively high concentrations for nearly all elements. It ranked among the top three for every element except manganese, where it was fifth, despite having the second highest partition coefficient, and silver, where overall concentrations were negligible, despite achieving the second highest capture-to-background ratio and the highest partition coefficient among all materials. This high performance, however, may primarily reflect elevated background levels for vanadium and nickel. Exact concentrations and *K*_*d*_ are listed in Figure  2.

Both gold and silver showed no significant capture. For silver, no material exhibited a partition ratio sufficient to indicate meaningful sorption. Although several materials displayed partition ratios for gold, the associated error variances exceeded 100% (Figure 2) and individual sample review usually indicates these results driven by a single outlier. Therefore, the apparent enrichment is likely attributable to background variability rather than genuine capture.

Nickel, copper, and manganese exhibited significantly higher captured concentrations, approximately an order of magnitude greater than the other elements, but it was limited to a small subset of materials. Specifically, elevated nickel was observed in orange peel and potato skin; copper in potato skin and peanut shell; and manganese in red tea and white birch. These materials contain naturally high background levels of the respective elements, either due to inherent composition or farming practices [15, 28].

Despite efforts to homogenize the samples, the biomaterials inherently display substantial variability in background concentrations on a mass basis, as these depend on the exposed surface characteristics of the original material. For example, the peanut shell samples captured 10–13 μg per test with a standard deviation of only 15%, but when normalised to sample mass, the variability increased to 50%. This suggests that there are latent variables impacting the results, such as individual particle porosity (Fig. 2).

Uranium exhibited the largest discrepancy between the filter-bags controls and free-contact samples; the element originally targeted in reference [22]. Garlic powder and dried kale showed limited capture within filter bags (31% and 17%, respectively) compared with their free -contact equivalents (88% and 87%). The third inter-batch control, peanut shell, showed only a small reduction (86% to 73%). In the case of the garlic, this loss was likely a consequence of fine particles passing through the filter, as the average final mass was only 49.1% of the initial. Dried kale also exhibited loss (82.5%), though primarily as dissolved organic carbon (DOC) compounds rather than visible particles. In both cases, these escaping materials likely acted to bind the elements in the wet phase, preventing further uptakes by the solid phase inside the bag. This might also occur with large ligands already present in the solution, such as Ca_2_(UO_2_)(CO_3_)_3(aq)_ [10]. These larger complexes would be less likely to be able to cross the barrier, even if they could physically fit.

Other than these two exceptions, all samples showed an increase in mass after contact. Observations indicate that this was primarily due to biofilm growth within the sample bags (see Table 4 for average increases), which acted as a sorptive matrix [3]. This finding demonstrates that a common concern of this approach, as mentioned in Li et al. (16), that the bio-materials are undergoing degradation under real-world conditions, reducing their effectiveness, is not a significant effect on the timescales of the tests and within a suitable barrier material.

**Table 4:** pH of final supernatant of the sample

Sample	Sample mass (g)	Post contact weight (g)	Mass change (%)	Variance (%)
Blackberry	1.13	1.26	113	8
Coffee grounds	1.86	2.37	130	9
Dried kale	1.04	0.85	83	5
Garlic powder	2.13	1.01	49	9
Peanut shell	0.94	1.52	169	18
Red tea leaf	2.67	2.85	107	2
Spring greens	0.76	1.05	155	27
White birch	1.19	1.80	158	4

The modelling work by McGowan, Degueldre, and Aiouache (20) and McGowan, et al., 21 also highlighted the susceptibility of the surfaces to changes in pH. Samples were analysed for pH, and compared with the *K*_*d*_ values the values are displayed in Table 5; however, no significant correlation was observed. This is likely due to the narrow pH range of the samples. Most materials equilibrated between 5.0 and 6.0, except for the grape and berry samples, which were as low as 4.0, likely due to the with high sugar contents driving increase fermentation reactions [17, 23]. It is unclear from this test if it is a factor in practice.

Overall, the results demonstrate that elemental capture using organic components is feasible. No single broad-spectrum material was effective across all tested elements, but some could be used for a subset. No signficiant material loss was observed over the timescales of the tests, and the observed pH shifts were consistent with fermentation processes [23].

A notable finding was that vanadium and uranium sorption did not appear to correlate, in contrast to the results by Sugo et al. (30), who observed good capture of both elements in the synthetic surfaces. This divergence is significant for the approach (Table 5).

**Table 5:** pH of sample

Without bag		With bag	
Dried kale	5.78	Blackberry	4.3
Garlic Powder	5.22	Coffee grounds	5.8
Orange peel	4.9	Garlic powder	5.6
Peanut shell	6.08	Dried kale	5.9
Potato skin	6.08	Peanut shell	5.9
Red grape	4.8	Spring greens	5
Vitira grape	4.25	Red tea leaf	5.4
White grape	6.07	White birch	6.2

The modelling study by McGowan et al. (20) proposed two potential mechanisms which could account for enhanced uranium deposition by organic materials: a single-electron redox reaction at − 0.8 V under higher pH conditions, or a double-electron reaction at − 1.2 V under lower pH. Both mechanisms yielded modelled *K*_*d*_ values consistent with those previously reported and reaffirmed in the presented results. When these potential shifts are applied to vanadium, using literature data [27], the corresponding redox states occur on opposite sides of a phase boundary. Under these conditions, vanadium would exist either as the absorbable form in the form of V^2+^ species if the shift was − 0.8 V, or as a neutral, non-sorbing form with the − 1.2 V shift. The observed results are consistent with the latter scenario, indicated by the lack of mutual sorption behaviour between vanadium and uranium, which would otherwise be expected to absorb.

## Conclusions

In general, these tests demonstrated that the batch liquid volume was was insufficient for consistent measurement, with capture rates of uranium being over 80% when the materials perform, indicating that increased volumes to greater than 50 ml per g of material will be required.

Furthermore, attempting to contain the materials within filtration proved impractical. Several of the elements are predominantly present as soluble complexes with ligands, such as carbonates, under the conditions of the tests, either naturally occurring, or those impelled by the test materials. Such complexes would be excluded from sorption, while containment does not effectively prevent migration of the organic material into solution, despite it’s reported characteristics being of the wrong scale to interfere. It remains unclear whether effective restriction could be achieved without inhibiting the underlying sorption mechanisms.

Among the new materials tested, only the White Birch bark exhibited promising *K*_*d*_ values; however, these results are not quantifiable due to limitations associated with the filtrants.

## Data Availability

No datasets were generated or analysed during the current study.
